# The Impact of Three Years of Targeted Indoor Residual Spraying with Pirimiphos-Methyl on Household Vector Abundance in a High Malaria Transmission Area of Northern Zambia

**DOI:** 10.4269/ajtmh.20-0537

**Published:** 2020-12-21

**Authors:** Marisa A. Hast, Jennifer C. Stevenson, Mbanga Muleba, Mike Chaponda, Jean-Bertin Kabuya, Modest Mulenga, Timothy Shields, William J. Moss, Douglas E. Norris, for the Southern and Central Africa International Centers of Excellence in Malaria Research

**Affiliations:** 1 Department of Epidemiology, Johns Hopkins Bloomberg School of Public Health, Baltimore, Maryland;; 2 W. Harry Feinstone Department of Molecular Microbiology and Immunology, Johns Hopkins Malaria Research Institute, Johns Hopkins Bloomberg School of Public Health, Baltimore, Maryland;; 3 Macha Research Trust, Choma, Zambia;; 4 The Tropical Diseases Research Centre, Ndola, Zambia;; 5 Department of Public Health, Michael Chilufya Sata School of Medicine, The Copperbelt University, Kitwe, Zambia

## Abstract

The global malaria burden has decreased substantially, but gains have been uneven both within and between countries. In Zambia, the malaria burden remains high in northern and eastern regions of the country. To effectively reduce malaria transmission in these areas, evidence-based intervention strategies are needed. Zambia’s National Malaria Control Centre conducted targeted indoor residual spraying (IRS) in 40 high-burden districts from 2014 to 2016 using the novel organophosphate insecticide pirimiphos-methyl. The Southern and Central Africa International Centers of Excellence for Malaria Research conducted an evaluation of the impact of the IRS campaign on household vector abundance in Nchelenge District, Luapula Province. From April 2012 to July 2017, field teams conducted indoor overnight vector collections from 25 to 30 households per month using Centers for Disease Control light traps. Changes in indoor anopheline counts before versus after IRS were assessed by species using negative binomial regression models with robust standard errors, controlling for geographic and climatological covariates. Counts of *Anopheles funestus* declined by approximately 50% in the study area and within areas targeted for IRS, and counts of *Anopheles gambiae* declined by approximately 40%. Within targeted areas, *An. funestus* counts declined more in sprayed households than in unsprayed households; however, this relationship was not observed for *An. gambiae*. The moderate decrease in indoor vector abundance indicates that IRS with pirimiphos-methyl is an effective vector control measure, but a more comprehensive package of interventions is needed with sufficient coverage to effectively reduce the malaria burden in this setting.

## INTRODUCTION

As a result of widespread scale-up of malaria control interventions, there has been a substantial decrease in the global malaria burden. From 2000 to 2015, global malaria incidence decreased by 41% and malaria mortality rates declined by 62%.^1^ However, these gains were uneven both within and between countries, and the rate of progress has slowed or reversed in some regions.^2^ This pattern is evident in parts of Zambia, where malaria remains a leading cause of child mortality despite significant improvements in malaria control.^2,3^

Between 2000 and 2008, inpatient malaria cases and deaths in Zambia declined by approximately two-thirds following a highly successful malaria control campaign that supported universal access to rapid diagnostic testing (RDT), artemisinin combination therapy, long-lasting insecticide-treated nets (LLINs), and expanded annual indoor residual spraying (IRS).^4^ However, whereas large gains were made in the capital Lusaka and the southern part of the country, a high burden of malaria continued largely unabated in northern and eastern regions of Zambia.^5^ In subsequent years, low transmission was maintained in the south, but declining funds and intervention effectiveness led to an increase in malaria cases in northeast Zambia in 2009, and cases continued to increase throughout much of the next decade.^5–8^ Most recently, the WHO estimated that there were 2.7 million malaria cases and 7,500 deaths in Zambia in 2018, which represents an overall increase in both cases and deaths since 2010.^2^ This heterogeneity of malaria control under the same intervention policy, with reversal of progress in northeastern Zambia, indicates a need to better understand intervention effectiveness in different epidemiologic settings.

Malaria transmission is dependent on the presence and abundance of mosquito vectors, and vector control is a key priority for Zambia’s national malaria control strategy.^2,9,10^ The main malaria vectors in northern Zambia are *Anopheles funestus* s.s. and *Anopheles gambiae* s.s., both of which are highly anthropophilic (feed on humans), endophagic (bite indoors), and endophilic (rest indoors).^4,11–15^ Because of these indoor behaviors, Zambia has prioritized indoor vector control strategies such as LLINs and IRS in this region. However, increasing resistance to pyrethroids, dichloro-diphenyl-trichloroethane, and carbamate insecticides has reduced the efficacy of these interventions.^7,11,16^ In response to this trend, a novel formulation of the organophosphate pirimiphos-methyl (Actellic^®^ 300CS, Syngenta, Basel, Switzerland) underwent susceptibility testing in 2013 and was demonstrated to be 100% effective against the malaria vectors in northern Zambia.^16,17^

In 2014, Zambia’s National Malaria Control Centre (NMCC) implemented an IRS campaign using pirimiphos-methyl in 40 high-burden districts in northern and central Zambia. Because of the increased cost of this insecticide, the NMCC elected to use a targeted rather than blanket IRS approach.^18^ Targeted IRS refers to an emerging strategy that focuses intervention activities on identified transmission hotspots to concentrate limited resources in areas that have the most impact in sustaining local transmission.^19^ Targeted IRS has been implemented in several countries in sub-Saharan Africa, and recent studies have shown promising results for reducing both parasite prevalence and vector densities in low- to medium-transmission areas.^20–26^ This campaign was one of the first examples of a targeted IRS strategy implemented in a high-transmission setting.

The impact of the targeted IRS campaign on human parasite prevalence was previously described for Nchelenge District, Luapula Province, a holoendemic area in northern Zambia.^27^ In brief, rainy season parasite prevalence declined by approximately 25% within areas targeted for IRS with pirimiphos-methyl but did not decline in neighboring unsprayed areas or during the dry season.^27^ This decrease in prevalence was smaller in magnitude and scope than expected, particularly given the high insecticidal effect of the novel compound on local vectors as indicated in earlier laboratory assays.^16,17^ The limited effectiveness of this IRS campaign and the continued high transmission in northern Zambia, despite active malaria control, highlight the need for comprehensive and evidence-based intervention strategies. To more directly investigate the effect of the targeted IRS strategy on malaria transmission and to help determine why parasite prevalence declined less than anticipated, it is necessary to examine the effect of the intervention on malaria vectors. To accomplish these objectives, this analysis aims to evaluate the direct impact of three consecutive years of targeted IRS with pirimiphos-methyl on household malaria vector abundance in Nchelenge District.

## METHODS

### National Malaria Control Centre targeted IRS campaign.

From 2008 to 2012, the U.S. President’s Malaria Initiative supported yearly IRS campaigns in Nchelenge District. Different formulations of pyrethroid insecticides were used from 2008 to 2010, and carbamate insecticides were used in 2011 and 2012 following identification of pyrethroid resistance.^28^ During this time, the strategy for vector control was to use IRS in urban and peri-urban areas and to distribute LLINs in rural areas. No IRS activities occurred in Nchelenge District in 2013.

In 2014, Zambian IRS activities in five provinces were transitioned to the Africa IRS program with Abt Associates as an implementing partner.^29,30^ Forty high-burden districts were identified in five provinces, and subdistrict areas were selected for targeted IRS with pirimiphos-methyl.^29,30^ Detailed methods for selection of subdistrict areas are described elsewhere.^18,19,31^ In brief, all structures in the 40 districts were enumerated using publicly available satellite images, and household clusters that had at least 25 households were identified within a 50-m buffer of each other. These clusters were linked to rural health center catchment areas and were ranked on predicted malaria burden based on population density and the health center’s reported malaria incidence. Clusters with fewer than 25 households or with insufficient household density were excluded, and high-ranking clusters were selected for targeted IRS. Aside from the 50-m buffer, there was no minimum or maximum distance between clusters that were or were not selected for targeted IRS.

Spray activities began in October 2014 with the goal of at least 85% coverage.^18^ Targeted IRS then occurred annually in 2015 and 2016, with targeting methodology adjusted to select larger targeted areas in fewer districts to connect geographically isolated targeted areas.^32,33^ During this time, a mass LLIN distribution occurred in Luapula Province from June to September 2014, and LLINs otherwise continued to be routinely distributed in antenatal and vaccination clinics.^28,34^

### Study site.

Data collection for this analysis was conducted in Nchelenge District, Luapula Province, by the Southern and Central Africa International Centers of Excellence for Malaria Research (ICEMR).^6,35^ The project uses active and passive surveillance to investigate heterogeneity in malaria burden across four distinct epidemiological settings.^35^ Nchelenge District is one of these surveillance sites and was among the districts selected for targeted IRS. The district is located in the marshlands of the Luapula River along the banks of Lake Mweru, which forms the border with Haut-Katanga Province of the Democratic Republic of the Congo (DRC). There are approximately 150,000 residents with an average of 4.7 people per household, and the majority of people live in mud huts with natural flooring, thatch roofs, and open eaves.^36^ Nchelenge District represents a setting of holoendemic malaria transmission.^7^ The prevalence of malaria by RDT averages approximately 50% in adults and 70% in school-age children, and the cumulative entomological inoculation rate is estimated to be 80–140 infective bites/person/year.^13,27,37,38^

The predominant malaria vectors in Nchelenge District are *An. gambiae* s.s. and *An. funestus* s.s., and the distribution of these vectors varies spatially throughout the year.^12,13^ *An. gambiae* breeding sites are typically shallow temporary pools such as wheel ruts and hoof prints, making this species dependent on rainfall, whereas *An. funestus* breeding sites are more frequently permanent bodies of water with emergent vegetation, such as marshlands and river banks.^14,15^ In Nchelenge District, there is a single rainy season from October to April; however, an extensive stream network throughout the region and plentiful swamplands along the lake and streambanks support year-round malaria transmission. *An. funestus* is found throughout the year in both lakeside and inland areas with a large peak in abundance in the dry season, and *An. gambiae* abundance peaks in smaller relative numbers primarily in lakeside areas shortly after the start of the rainy season.^12,13,39^

### Data collection.

The Southern and Central Africa ICEMR has conducted active surveillance in Nchelenge District since April 2012. Households in the study area were enumerated using Quickbird™ satellite images (DigitalGlobe Services, Denver, CO). A 1 × 1-km grid was overlaid on the study area, and grid quadrants were selected using spatially balanced random sampling to ensure that households were represented from a range of ecological settings in the study area. Within each quadrant, households were randomly selected using population proportional to size sampling. Each month, between one and six households were selected per grid quadrant. Households were recruited into longitudinal or cross-sectional cohorts, with sampling alternating between cohorts every other month. Longitudinal cohorts comprised 25–30 households visited bimonthly six times over a year and then replaced with a new longitudinal cohort. In the alternating cross-sectional months, approximately 25 new households were recruited and were visited only once. Household selection was independent of the targeted IRS intervention or other malaria control activities.

At each study visit, household coordinates were recorded, and mosquitoes were collected overnight using CDC miniature light traps (John W. Hock, Ltd., Gainesville, FL). Traps were placed indoors in a sleeping area adjacent to an occupied LLIN. Household members were instructed to turn traps on at 18:00, to close the collection bags, and then to turn the traps off at 6:00 the following morning, after which staff collected the traps. Consenting household members were administered a questionnaire on demographic information, household structure, household water source, reported LLIN use, and history of household IRS. Mosquitoes were killed by freezing, identified morphologically to genus and sex, and enumerated, and the anophelines were stored individually dry on silica at the field station in Kashikishi township. The mosquito samples were transported to the Tropical Diseases Research Centre headquarters in Ndola once per month for final laboratory identification using standard morphological keys.^40,41^ Additional details of study methods are described elsewhere.^13^

### Data management.

**Figure 1.**
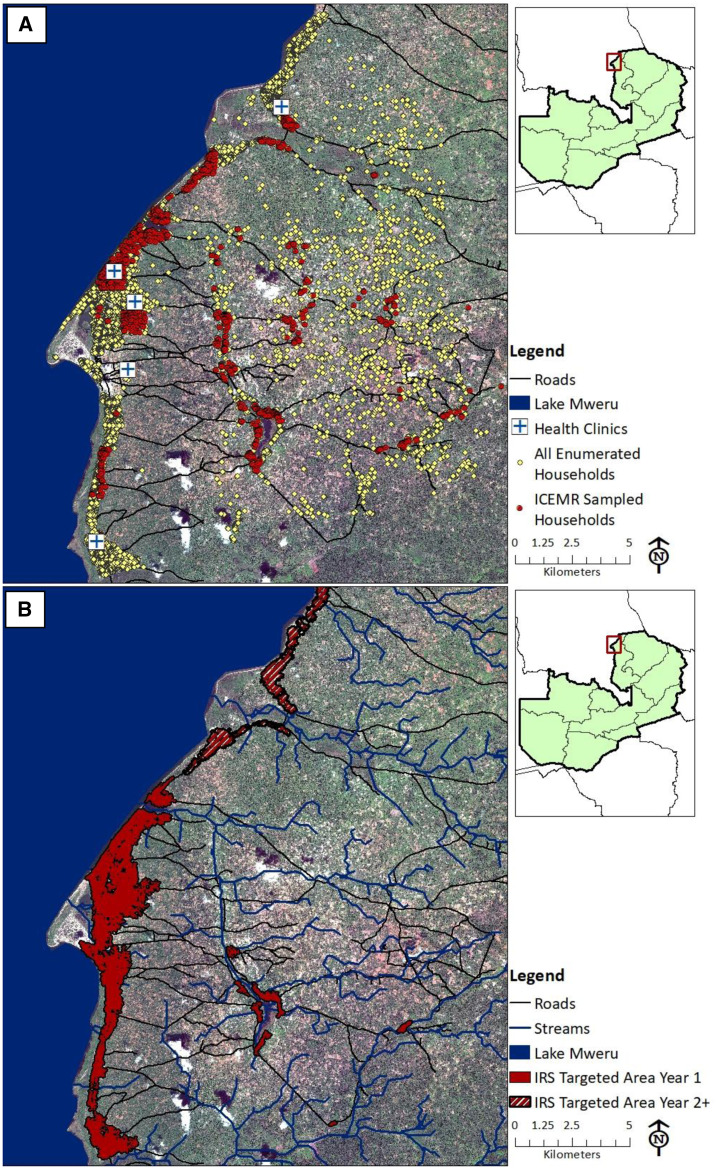
(**A**) Nchelenge District sampled and enumerated households April 2012–July 2017; (**B**) areas in Nchelenge District targeted for indoor residual spraying (IRS) in year 1 (2014) and years 2 and 3 (2015 and 2016). Reprinted with permission from Hast et al.^27^ This figure appears in color at www.ajtmh.org.

Data collected from participating households were uploaded into REDCap secure file-sharing software.^42^ Participating households were plotted in ArcGIS version 10.2 (ESRI, Redlands, CA) (Figure 1A), and population density at each household was calculated as the total number of enumerated households within a 500-m buffer. Geographic variables were created from previously developed georeferenced raster and shapefiles for roads, stream networks, elevation, slope, and normalized difference vegetation index (NDVI) for the study area.^43^ Streams were categorized using the Strahler classification system, in which two small category 1 streams join to form a category 2 stream, two category 2 streams join to form a category 3 stream, and so on.^44^ Distances to Lake Mweru, health centers, roads, and category 1–4 streams were calculated for each household. Based on a natural break in household density, households were categorized as lakeside (rather than inland) if they were within 3 km of Lake Mweru. Residence in the area targeted for spraying in each year was determined using shapefiles provided by the NGO partner Akros.^19^

Meteorological and hydrological data were collected from a HOBO Micro Station (Onset Computer Corporation, Bourne, MA) located near the field station and from the African Flood and Drought Monitor online tool.^45,46^ Variables collected included rainfall in mm/day, evaporation in mm/day, minimum and maximum daily temperature in °C, wind speed in m/s, streamflow in m^3^/s, and percent soil moisture. The start and the end of the rainy season each year was defined as the first and last weeks in which the average rainfall exceed 1 mm. Sensitivity analyses using different cutoffs and time intervals were performed to ensure that this definition best represented the epidemiologic and entomologic relationships in this region.

### Statistical analysis.

Data for this analysis were collected between April 2012 and July 2017. The primary outcome of interest was the change in household vector abundance by species before versus after the implementation of targeted IRS with pirimiphos-methyl. As a result of differing malaria transmission dynamics and vector species distribution between lakeside and inland regions of Nchelenge District, as well as the disproportionate targeting of IRS to lakeside areas (Figure 1B), a direct comparison between sprayed and unsprayed areas would be biased and was not conducted.

Data were analyzed using STATA 13.1 (Stata-Corporation, College Station, TX) and *R* 3.4.2 (R Core Team, Vienna, Austria). All entomological, epidemiological, and climatological data collected during this time were merged by household and day. Vector counts did not differ significantly between longitudinal and cross-sectional cohorts, so vector data were included from all enrolled households. Bivariate and multivariate models were developed by species using negative binomial models with robust standard errors to account for overdispersion.^47,48^ The unit of analysis was the household, with indoor vector counts by species as the outcome. Generalized estimating equations were used to account for repeat visits to longitudinal households.^49,50^ Models were developed for both targeted areas only and for the overall study area. Models were not stratified by season because of the high dispersion in the data and a lack of power to run stratified analyses.

Preliminary multivariate models were developed using all covariates significant at the *P* = 0.1 level in bivariate comparisons or identified as relevant in previous studies.^13,39^ To help account for secular trends, a cross-correlation approach was adapted for meteorological and hydrological variables to identify the most etiologically relevant time period to predict household vector counts.^39,51,52^ In brief, mean values of each variable (i.e., rainfall) were calculated at intervals of 1–12 weeks and lags of 1–12 weeks before each day of data collection, returning 144 potential covariates for each climatological factor. A preliminary list of the most predictive climate variables was identified using random forest algorithms,^53,54^ and then final model selection was conducted using all relevant variables by stepwise regression and AIC optimization methods.^55,56^

In models restricted to targeted areas, secondary analyses were conducted to determine the indirect effects of the IRS intervention on household vector abundance. Households in targeted areas were stratified by self-reported history of household IRS with pirimiphos-methyl, and the change in household vector abundance by species before versus after the intervention was determined separately for sprayed and unsprayed households.

A difference-in-differences analysis was also conducted to further account for secular trends. This analysis assumes that, although baseline vector abundance by species may have been different in sprayed and unsprayed areas as a result of ecological differences or other factors, the proportionate change in vector abundance over time would be equal if the IRS campaign had not occurred. The value of an interaction term in this model (before versus after IRS*targeted versus untargeted area) is interpreted as the ratio of risk ratios, or the ratio of the change in vector abundance in targeted areas over the change in untargeted areas.

### Sensitivity analyses.

As a result of the highly skewed nature of the data, several sensitivity analyses were conducted on the models restricted to targeted areas. Initially, households with vector counts ≥ 3 SDs from the mean for each species were removed from models. It was hypothesized that these outliers could unduly impact models and mask underlying effects. In a second sensitivity analysis, participants living in the small isolated sprayed areas found in the inland region of the study area (Figure 1B) were excluded under the hypothesis that these targeted areas were of insufficient spatial area and/or contained too few households to produce an effective result.

## RESULTS

### Characteristics of targeted IRS.

Targeted IRS activities with pirimiphos-methyl started between September and October each year and ran for 7–10 weeks.^18,32,33^ As a result of low population density in inland rural areas, targeted areas were primarily located in the peri-urban lakeside region (Figure 1B). The number of targeted households in Nchelenge District was 18,315 in 2014 and increased to approximately 26,000 in 2015 and 2016 with the addition of more targeted areas.^32,33,57^ In official reports, 17,367 households were sprayed in Nchelenge District in 2014, 24,219 were sprayed in 2015, and 26,027 were sprayed in 2016.^29,32,33,36^ This translates to approximately 49%, 66%, and 69% coverage of all households in Nchelenge District, accounting for 2.9% population growth per year.^36^ By self-report, 54% of surveyed households in targeted areas and 41% of all surveyed households reported that they were sprayed over the 3 years of IRS.^27^ Quality assurance activities conducted in five sentinel sites showed 100% mortality of *An. funestus* in cone bioassays 24 hours after spraying, declining to less than 80% mortality after 5 months.^28^

### Vector species composition.

**Figure 2.**
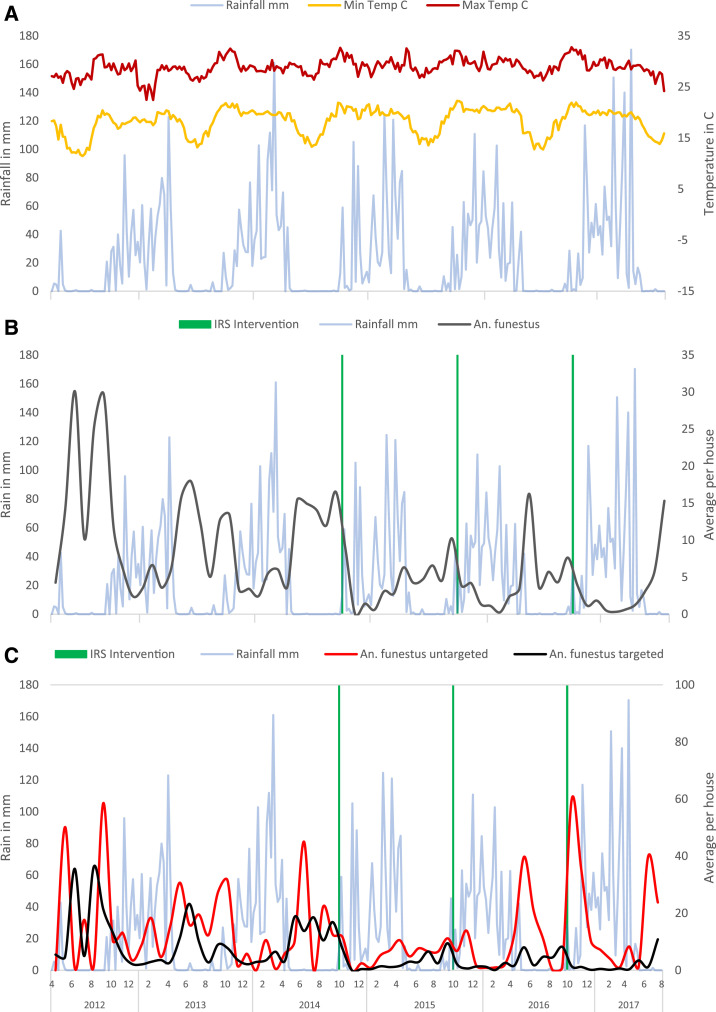
Time series of (**A**) weather patterns, (**B**) *Anopheles funestus* counts in the whole study area, and (**C**) *Anopheles funestus* counts in sprayed vs. unsprayed areas. This figure appears in color at www.ajtmh.org.

**Figure 3.**
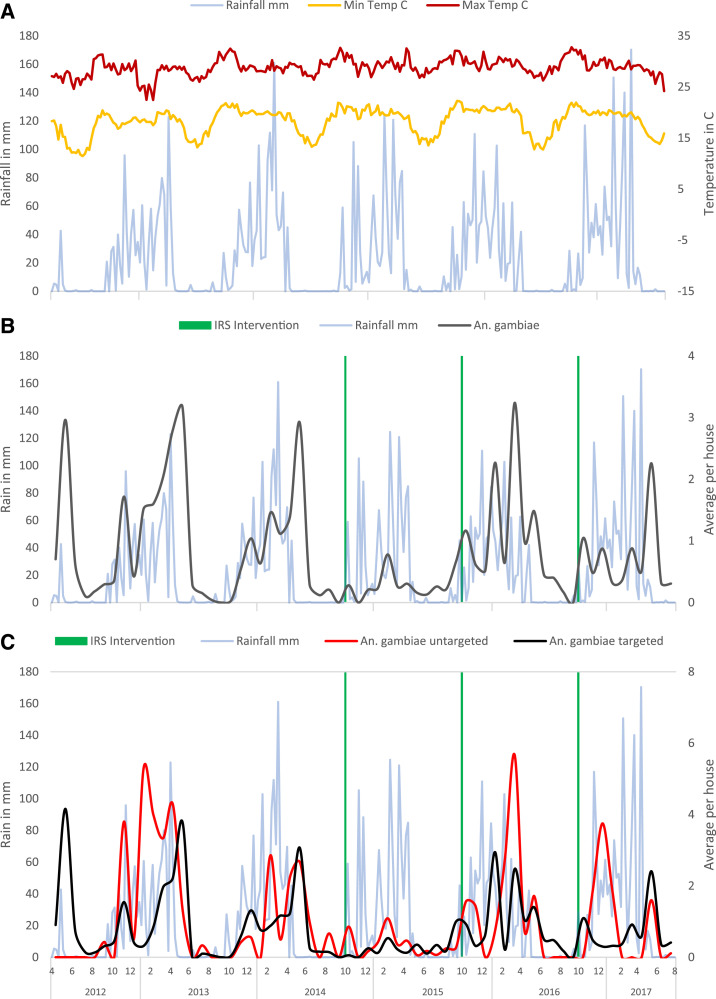
Time series of (**A**) weather patterns, (**B**) *Anopheles gambiae* counts in the whole study area, and (**C**) *Anopheles gambiae* counts in sprayed vs. unsprayed areas. This figure appears in color at www.ajtmh.org.

From April 2012 to July 2017, 13,780 female anopheline mosquitoes were collected from 1,724 visits (trap-nights) to 1,084 cross-sectional and longitudinal households in 39 grid quadrants. These included 12,365 *An. funestus*, 1,371 *An. gambiae*, and 44 anophelines of other species.^39^ *An. funestus* had the highest vector counts throughout the year, with a peak in abundance in the dry season (Figure 2). *An. gambiae* counts peaked in the rainy season and were rare or absent in the dry season (Figure 3). Across all visits, households had an average of 7.2 and a median of 0 *An. funestus* (range = 0–226, interquartile range [IQR] 0–2), and an average of 0.8 and a median of 0 *An. gambiae* (range = 0–35, IQR 0–0). As previously described, the distribution of household vector counts was highly skewed, with 53% of household visits yielding no mosquitoes and 5% of household visits yielding 50–230 female anophelines (Supplemental Figure S1).^39^ By species, 60.7% of household visits yielded no *An. funestus*, and 77.5% of household visits yielded no *An. gambiae.*

### Impact of targeted IRS on household vector counts.

#### Impact of targeted IRS in the overall study area.

Over the entire study area, an average of 10.6 *An. funestus* and 0.96 *An. gambiae* were collected per household visit (trap-night) before the IRS campaign, which declined to 4.2 and 0.65 per visit, respectively, after IRS with pirimiphos-methyl was initiated (Table 1). Because more than half of households had no *Anopheles* mosquitoes collected, the median values did not change; however, in Wilcoxon rank-sum tests, *An. funestus* counts were significantly lower after the intervention in unadjusted analyses (*P* = 0.01) but *An. gambiae* counts were not (*P* = 0.7). Similarly, in bivariate negative binomial models, which control for clustering within household but do not account for other covariates, there was an overall 58% decrease in household *An. funestus* counts (incidence rate ratio [IRR] = 0.42; 95% CI = 0.31–0.59) and a 31% decrease in household *An. gambiae* counts (IRR = 0.69; 95% CI = 0.50–0.96) after targeted IRS.

**Table 1:** Unadjusted average counts of *An. funestus* and *An. gambiae* per household visit before and after IRS with pirimiphos-methyl

	*An. funestus*	*P*-value	*An. gambiae*	*P*-value
Total study area				
Pre-IRS	10.6	0.01	0.96	0.7
Post-IRS	4.2	–	0.65	–
Sprayed areas				
Pre-IRS	9.4	0.007	0.90	0.4
Post-IRS	2.6	–	0.64	–
Unsprayed areas				
Pre-IRS	15.3	0.3	1.3	0.4
Post-IRS	8.6	–	0.69	–

*An. funestus* = *Anopheles funestus*; *An. gambiae* = *Anopheles gambiae*; IRS = indoor residual spraying; *P*-values are from Wilcoxon rank-sum tests.

In multivariate models for the entire study area controlling for all geographic and climate variables, there was a 51% decline in *An. funestus* counts per household (IRR = 0.49; 95% CI = 0.29–0.82) and a 40% decline in *An. gambiae* counts per household (IRR = 0.60; 95% CI = 0.44–0.80) after the initiation of targeted IRS with pirimiphos-methyl (Table 2). These results indicate that there was an overall decline in household vector counts throughout the study area following the IRS campaign.

**Table 2:** Negative binomial multivariate models of the impact of targeted IRS with pirimiphos-methyl on *An. funestus* and *An. gambiae* counts per household over the entire study area, using robust standard errors and generalized estimating equations clustered by household, *N* = 1,724

	*An. funestus*			*An. gambiae*		
	IRR	95% CI	*P*-value	IRR	95% CI	*P*-value
Post-IRS	0.49	(0.30, 0.82)	*0.007*	0.60	(0.44, 0.80)	*0.001*
HH within 500 m (by 100 HH)	0.66	(0.54, 0.81)	< *0.001*	0.82	(0.75, 0.89)	< *0.001*
Elevation (by 10 m)	0.53	(0.46, 0.61)	< *0.001*	–	–	–
Slope	0.88	(0.80, 0.97)	*0.007*	–	–	–
NDVI (by 10%)	1.24	(1.03, 1.49)	*0.02*	–	–	–
Lakeside	0.24	(0.14, 0.41)	< *0.001*	0.29	(0.16, 0.50)	< *0.001*
Distance from Lake Mweru (in 1,000 km)	–		–	0.83	(0.77, 0.89)	< *0.001*
Distance from roads (in 100 m)	0.80	(0.74, 0.86)	< *0.001*	0.82	(0.75, 0.91)	< *0.001*
Distance from cat. 1 streams (in km)	0.49	(0.31, 0.98)	*0.003*	0.56	(0.41, 0.78)	< *0.001*
Lagged rainfall (by 10 mm) *	0.27	(0.16, 0.48)	*< 0.001*	–	–	–
Lagged rainfall (by 10 mm) †	0.62	(0.39, 0.96)	*0.03*	–	–	–
Lagged rainfall (by 10 mm) ‡	–	–	–	0.67	(0.48, 0.94)	*0.02*
Lagged rainfall (by 10 mm) §	–	–	–	2.33	(1.40, 3.86)	*0.001*
Lagged maximum temperature (in C°) *	1.08	(1.01, 1.16)	*0.03*	–	–	–
Lagged maximum temperature (in C°)∥	0.80	(0.69, 0.92)	*0.003*	–	–	–
Lagged maximum temperature (in C°) ¶	–	–	–	0.81	(0.73, 0.90)	*< 0.001*
Lagged minimum temperature (in C°) ¶	–	–	–	1.30	(1.20, 1.41)	*< 0.001*

*An. funestus* = *Anopheles funestus*; *An. gambiae* = *Anopheles gambiae*; IRS = indoor residual spraying; IRR = incidence rate ratio; HH = household; NDVI = normalized difference vegetation index. Entries in italics have *P*-values < 0.05.

* Interval = 2 weeks, lag = 2 weeks.

† Interval = 2 weeks, lag = 4 weeks.

‡ Interval = 1 week, lag = 2 weeks.

§ Interval = 7 weeks, lag = 3 weeks.

∥ Interval = 8 weeks, lag = 4 weeks.

¶ Interval = 4 weeks, lag = 3 weeks.

#### Impact of targeted IRS in sprayed areas.

In analyses restricted to areas targeted for IRS, there was an average of 9.4 *An. funestus* and 0.90 *An. gambiae* collected per household visit (trap-night) before the IRS campaign, which declined to 3.2 and 0.64, respectively, after IRS with pirimiphos-methyl. Similar to the results at the study area level, this decline was significant in Wilcoxon rank-sum tests for *An. funestus* (*P* = 0.007) but not for *An. gambiae* (*P* = 0.4) (Table 1). In bivariate negative binomial models adjusting for clustering within household but not for other covariates, *An. funestus* counts declined in sprayed areas by 68% after the IRS intervention (IRR = 0.32; 95% CI = 0.22–0.48), but declines in *An. gambiae* counts were not statistically significant (IRR = 0.73; 95% CI = 0.49–1.08).

**Figure 4.**
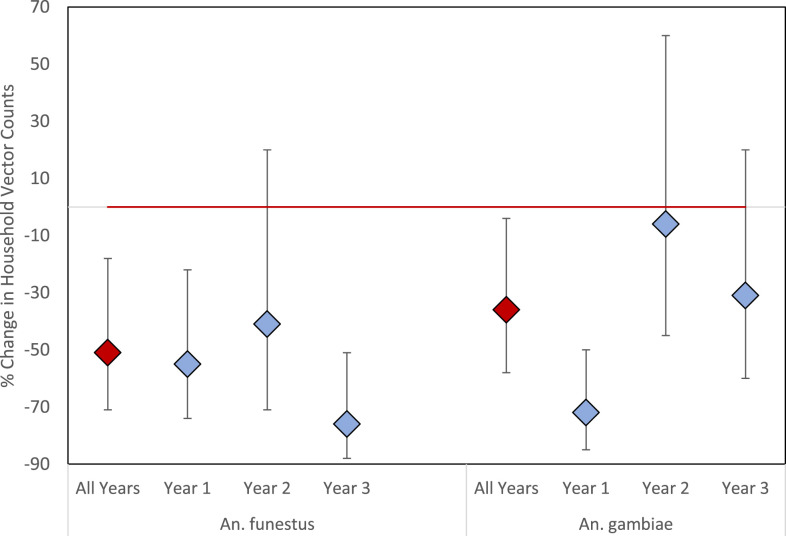
Reduction in household vector counts in Nchelenge District, Zambia, by year compared with pre-indoor residual spraying time period. This figure appears in color at www.ajtmh.org.

In final multivariate models restricted to the sprayed area and controlling for all geographic and climatological covariates, there was a 51% decrease in *An. funestus* counts (IRR = 0.49; 95% CI = 0.29–0.82) and a 36% decrease in *An. gambiae* counts (IRR = 0.64; 95% CI = 0.42–0.96) over 3 years of IRS with pirimiphos-methyl. (Table 3). This was similar to the decline in the overall study area. For *An. funestus*, there were no significant differences from year to year, but there was a nonsignificant trend toward a larger change in year 3, with a 76% reduction compared with a 55% and 41% reduction in years 1 and 2, respectively (Figure 4). The impact on *An. gambiae* counts was significantly higher in the first year of the IRS campaign, which had a 72% reduction, compared with the second and third years, which had 6% and 31% reductions, respectively (Figure 4). The addition of covariates and climatological factors improved the fit of the model but did not substantially change point estimates from unadjusted analyses (Supplemental Figure S2).

**Table 3:** Negative binomial multivariate models of the impact of targeted IRS with pirimiphos-methyl on *An. funestus* and *An. gambiae* counts per household within the areas targeted for spraying, using robust standard errors and generalized estimating equations clustered by household, *N* = 1,271

	*An. funestus*			*An. gambiae*		
	IRR	95% CI	*P*-value	IRR	95% CI	*P*-value
Post-IRS	0.49	(0.29, 0.81)	*0.005*	0.64	(0.42, 0.96)	*0.03*
Open water source	–	–	–	1.41	(1.02, 1.95)	*0.04*
HH within 500 m (by 100 HH)	0.60	(0.51, 0.70)	*< 0.001*	0.81	(0.75, 0.88)	*< 0.001*
Elevation (by 10 m)	0.43	(0.33, 0.55)	*< 0.001*	–	–	–
Slope	0.82	(0.73, 0.92)	*0.001*	–	–	–
NDVI (by 10%)	1.29	(0.99, 1.66)	0.06	1.23	(1.00, 1.52)	*0.05*
Distance from Lake Mweru (1,000 in km)	1.12	(1.03, 1.21)	*0.005*	–	–	–
Distance from cat. 1 streams (in km)	0.55	(0.31, 0.98)	*0.04*	0.49	(0.34, 0.73)	*< 0.001*
Distance from cat. 4 streams (in km)	1.42	(1.18, 1.71)	*< 0.001*	–	–	–
Lagged rainfall (by 10 mm)*	0.24	(0.13, 0.47)	*< 0.001*	0.68	(0.47, 0.99)	*0.05*
Lagged rainfall (by 10 mm)†	–	–	–	3.96	(1.88, 8.37)	*0.001*
Lagged maximum temperature (in °C)‡	1.14	(1.03, 1.26)	*0.01*	–	–	–
Lagged maximum temperature (in °C)§	0.79	(0.66, 0.94)	*0.007*	–	–	–
Lagged maximum temperature (in °C)∥	–	–	–	0.79	(0.67, 0.92)	*0.002*
Lagged minimum temperature (in °C)∥	–	–	–	1.32	(1.18, 1.48)	*< 0.001*

*An. funestus* = *Anopheles funestus*; *An. gambiae* = *Anopheles gambiae*; HH = household; IRR = incidence rate ratio; IRS = indoor residual spraying; NDVI = normalized difference vegetation index.

* Interval = 2 weeks, lag = 2 weeks.

† Interval = 10 weeks, lag = 4 weeks.

‡ Interval = 1 week, lag = 2 weeks.

§ Interval = 8 weeks, lag = 3 weeks.

∥ Interval = 7 weeks, lag = 2 weeks.

#### Indirect effects of IRS.

To investigate direct and indirect effects of the IRS intervention on household vector counts, models restricted to the sprayed area were further stratified by self-reported history of IRS. In fully adjusted multivariate models, *An. funestus* counts decreased 67% in households that reported IRS with pirimiphos-methyl (IRR = 0.33; 95% CI = 0.20–0.52) compared with household counts before the IRS campaign, but declines were not statistically significant in households within the sprayed area that reported no history of IRS (IRR = 0.64; 95% CI = 0.32–1.27). Unexpectedly, declines in *An. gambiae* counts among households that reported IRS with pirimiphos-methyl were not statistically significant (IRR = 0.74; 95% CI = 0.46–1.18); conversely, households within the sprayed area with no reported history of IRS had a 52% decline in *An. gambiae* counts compared with household counts before the IRS campaign (IRR = 0.48; 95% CI = 0.29–0.78).

#### Difference-in-differences analysis.

The difference-in-difference analysis compared the change in vector counts within the sprayed area with the change in counts in the unsprayed area. Negative binomial models for this method included an interaction term which represented the change in vector abundance after the IRS intervention in sprayed areas divided by the change in vector abundance in unsprayed areas, or the ratio of risk ratios. For both species, vector counts were significantly lower after IRS. However, this decrease was only larger in the sprayed area for *An. funestus*, and the interaction term signifying the ratio of risk ratios was not significant for either species (*An. funestus* = 0.67, 95% CI = 0.31–1.42; *An. gambiae* = 1.2, 95% CI = 0.62–2.40). These results indicate that there was a study area–wide reduction in vector counts, that this reduction was qualitatively larger for *An. funestus* within sprayed areas compared with unsprayed areas, but that the change in vector counts was not significantly different between sprayed and unsprayed areas for either species.

### Impact of covariates on vector abundance.

In addition to the IRS campaign, several other household-level, geographic, and climatological factors were associated with household vector counts (Tables 2 and 3). These associations are generally consistent with previous results,^39^ and are presented in detail in the Supplement Material.

### Sensitivity analyses.

Sensitivity analyses were run on models restricted to targeted areas to account for the influence of outliers and geographically isolated targeted areas. In brief, results of sensitivity analyses were consistent with those of standard models, indicating that results are not biased by outliers. More details on results of sensitivity analyses can be found in the Supplement Material.

## DISCUSSION

After 3 years of targeted IRS with pirimiphos-methyl, there was a decrease in household vector abundance in Nchelenge District, Zambia. *An. funestus* counts declined by approximately 50% over the entire study area, including both the sprayed and unsprayed areas, and this value was consistent across analyses. Although there was a slightly larger decrease in the sprayed area than in the unsprayed area, the results of the difference-in-differences analysis indicate that this difference was not statistically significant. However, within the targeted area, the decline in *An. funestus* vector abundance was twice as large in households that reported IRS as in households that did not report IRS.

Similarly, *An. gambiae* counts per household decreased after the IRS intervention by 40% in the entire study area and by approximately 36% in the area targeted for spraying. However, this result was less consistent across analyses, likely because of small sample sizes and resulting wide CIs. The decline in the unsprayed area was larger than that in the sprayed area, a surprising result, but the results of the difference-in-differences analysis again indicate that this difference was not statistically significant. When households within the targeted areas were stratified by reported history of IRS, households that had not been sprayed unexpectedly showed a larger decline in vector abundance after the intervention than households that had been sprayed, and this outcome was consistent across multiple sensitivity analyses. There was also a large degree of variability between years in the impact of targeted IRS on *An. gambiae*. Although specific causes of these differences could not be ascertained as a result of low sample sizes, the lower impact in year 2 is consistent with a lower impact of targeted IRS on rainy season parasite prevalence in unadjusted analyses.^27^

These results generally indicate that there was a significant and moderate reduction in indoor *An. funestus* and *An. gambiae* abundance in the study area following targeted IRS with pirimiphos-methyl, but that the cause of this decline is likely multifactorial and may not be wholly due to the intervention. Although it is feasible that the IRS intervention reduced vector counts district wide because of long flight and dispersal distances of host-seeking *Anopheles* mosquitoes,^58,59^ unsprayed households sampled for this study were often 5–10 km away from the sprayed area, and it has yet to be established whether mosquitoes disperse this far in this setting. Mark–release–recapture studies have shown a wide range of mean flight distance for different anopheline species ranging from less than 100 m to more than 12 km.^60^ The absence of a larger effect in the sprayed area than in the unsprayed area is surprising, particularly for *An*. *gambiae*, as is the absence of a significant decline in *An*. *gambiae* counts in sprayed households in stratified analyses. These results additionally contrast with the previously described epidemiologic impact of the targeted IRS campaign in Nchelenge District, which showed a reduction in malaria parasite prevalence in targeted areas but not in the overall study area.^27^

These unexpected findings may be explained in part by the high variability in the vector data and the large number of zero counts, which may reduce model stability and may increase the impact of outliers. This is particularly true for *An. gambiae* counts, for which nearly 80% of trap-nights yielded no captured *An*. *gambiae*. Given these challenges, it is possible that these models lack the power to conclusively demonstrate the isolated effect of the intervention on vector abundance in Nchelenge District as compared with other factors, particularly in stratified models. Still, the demonstrated reduction in vector counts is encouraging. A combination of low sample sizes in unsprayed areas and the need to reduce vector abundance disproportionately to see a reduction in malaria transmission could explain the discordance with epidemiologic results. Vectorial capacity and entomologic inoculation rate must be reduced substantially to reduce transmission in high-transmission areas.^61–63^ As a result of the higher baseline density of vectors in inland areas before the intervention, it is plausible that reductions in vector counts in unsprayed regions were genuine but were not great enough to result in significant declines in parasite prevalence.

These results also highlight the continuing challenges of malaria control in this high-transmission region. Although overall vector abundance declined after targeted IRS, high vector counts continued to be collected from both sprayed and unsprayed households throughout Nchelenge District, with counts of up to 93 *An. funestus* and 35 *An. gambiae* collected from single sprayed households in a night 6 months after the intervention. These findings indicate some potential limitations of the current IRS strategy for this setting. Although freshly sprayed pirimiphos-methyl was highly effective against local malaria vectors, studies have shown that this formulation produces only 5–8 months of insecticidal activity, with a particularly short duration of efficacy on the natural walls common in northern Zambia.^17,33,64–66^ Since Nchelenge District experiences year-round transmission, a single annual application of insecticide at the start of the rainy season in subdistrict areas will likely not be sufficient to interrupt transmission. Furthermore, only 54% of participating households in IRS-targeted areas reported that their house had been sprayed, which corresponds to just 55% of targeted participants and 42% of all participants in the study area. The 2015 Malaria Indicator Survey similarly reported that only 32% of households in Luapula Province had received IRS in the past year.^67^ This level of coverage is substantially lower than the goal of 85% and likely attenuated the impact of the IRS campaign.^18^ Finally, the population in Nchelenge District is highly mobile, and movement between the targeted and untargeted areas or across the border to the neighboring (unsprayed) DRC may increase the risk of transmission between people and mosquitoes.^68^

Given these substantial barriers and challenges, the question remains of how best to implement malaria control activities in Nchelenge District and similar regions with high transmission. The persistent high abundance of mosquito vectors in the district in addition to high baseline parasite prevalence, a large asymptomatic reservoir, and a highly rural dispersed population indicate that additional intervention strategies may be needed. The moderate impact of targeted IRS on both indoor vector abundance and parasite prevalence indicates that IRS continues to be a valuable intervention; however, the limited efficacy for both outcomes indicates that a targeted IRS strategy is likely not suitable for this region or other high-transmission areas. Mathematical models have also demonstrated that once-yearly IRS is insufficient to substantially reduce malaria prevalence in high-transmission areas, and dry season malaria transmission has been implicated in failure of malaria control even in areas with low transmission or a single transmission season.^69–71^ This indicates that the high degree of dry season transmission in Nchelenge District will undermine intervention effectiveness if malaria control measures are concentrated on rainy season transmission only. Furthermore, recent studies have additionally shown that *An*. *funestus* and *An*. *gambiae* may have substantial outdoor-biting behavior if people are outside during peak hours,^63,71–73^ thus limiting the effectiveness of many vector control activities.

Based on these findings, it is recommended that IRS with an effective insecticide be conducted in Nchelenge District twice per year at > 85% coverage along with the full suite of other malaria control activities, including LLIN distribution and improved case management. Ideally, the areas targeted for IRS should also be expanded to include the entire population of the district. However, if very remote areas are not accessible for IRS programs because of road conditions or financial limitations, alternative insecticidal interventions could be considered such as piperonyl butoxide bed nets. Piperonyl butoxide nets have been shown to be effective at reducing malaria transmission in the absence of IRS and to be superior to standard LLINs in areas of high pyrethroid resistance such as Nchelenge District.^74,75^ Additional interventions could also be explored to further reduce transmission, such as improved housing or vector control methods that target outdoor-biting or resting mosquitoes. Household construction particularly has been a consistent predictor of malaria and vector abundance across studies,^76–81^ and interventions to reduce mosquito entry could be impactful in future malaria control efforts. For example, interventions to add screens or close eaves have been shown to successfully reduce household entry by *An*. *gambiae.*^81–83^

This study had several limitations. As a result of the high number of zero vector counts and overdispersion of the data, the study had limited power to investigate the impact of the IRS campaign at finer spatial and temporal scales. The ICEMR study was designed as a surveillance system and therefore was not powered for specific investigative questions, particularly regarding the vector surveillance arm which collects only 25 data points per month. A larger sample size would aid in performing intervention evaluations with vector data, but this is extremely labor- and laboratory-intensive and was not possible for the present study nor programmatically feasible for ongoing surveillance. Future studies on the impact of interventions on household vector abundance would benefit from a higher number of households sampled per month and could consider other methods of vector collection, such as human landing catches.

Despite these issues, Nchelenge District remains one of the only study sites in the world with this length of longitudinal malaria vector data, so these conclusions continue to have great value for malaria programs despite their limitations. As the time series for the ICEMR study continues to increase over time, there will be increased power to investigate these types of questions moving forward.

## CONCLUSION

Three years of targeted IRS in Nchelenge District was associated with significant reductions in indoor vector abundance for *An*. *funestus* and *An*. *gambiae*. These reductions were observed in both areas targeted for IRS and in the overall study area. However, a lack of differential impact in areas that were and were not targeted for IRS indicate that the causes for these declines might be multifactorial and not due exclusively to the IRS campaign. Additional research is needed to determine the most effective interventions for vector control in this setting, and substantial investment continues to be needed in this region to achieve successful malaria control.

## Supplemental material

Supplemental materials
