# Supplementary material for: The Impact of Three Years of Targeted Indoor Residual Spraying with Pirimiphos-Methyl on Household Vector Abundance in a High Malaria Transmission Area of Northern Zambia
Source: Am J Trop Med Hyg. 2020 Dec 21;104(2):683–94. doi: 10.4269/ajtmh.20-0537 (PMC7866301; doi:10.4269/ajtmh.20-0537)
Supplement: Supplementary file 1 [file tpmd200537.SD1.pdf]

SUPPLEMENTAL MATERIAL

Impact of Covariates on Vector Abundance

In addition to the IRS campaign, several other factors were associated with household vector counts (Tables 2, 3). Consistent with previous results,[1] higher household density and increasing distance from category 1 streams were associated with lower vector abundance for both species, and increasing normalized difference vegetation index (NDVI) was associated with increased vector abundance. For *An. funestus*, higher elevation and steeper slope were associated with lower vector counts, and increasing distance from Lake Mweru and category 4 streams were associated with higher vector counts. For *An. gambiae*, household use of an open water source was associated with higher vector abundance. The climatological variables that best predicted vector abundance were lagged rainfall and temperature at various time intervals and lags (Tables 2, 3). Increased rainfall had a strongly negative impact on *An. funestus* counts, while *An. gambiae* counts were, conversely, strongly positively associated with rainfall except at very short time lags. *An. funestus* counts were positively associated with maximum temperature at short time intervals and were negatively associated with maximum temperature at longer time intervals, while *An. gambiae* counts were positively associated with minimum temperature but negatively associated with maximum temperature. These results indicate that the relationship between vector abundance and temperature is complex and may vary depending on species and stage of life cycle.

Sensitivity analyses

Sensitivity analyses were run on models restricted to targeted areas to account for the influence of outliers and geographically isolated targeted areas. Excluding households with vector counts ≥3 standard deviations from the mean removed all household visits with ≥75 *An. funestus* (40 households, 2.3% of total) or ≥9 *An. gambiae* (33 households, 1.9% of total) in their respective models. Excluding

households in the small isolated inland targeted areas removed 198 household visits from analyses, or 11.8% of total household visits. Households in these inland targeted areas did have higher average counts of both *An. funestus* and *An gambiae* after the IRS campaign compared with households in the larger lakeside targeted areas (mean *An. funestus* = 9.7 vs 1.6, P<0.001; mean *An. gambiae* = 1.9 vs 0.46, P<0.001). However, in all sensitivity analyses, there were no qualitative differences in the measured impact of the targeted IRS campaign for either species (Tables S1, S2). This result was consistent in standard models, models of indirect effects, and difference-in-differences analyses. Due to these conclusions, the standard models with all data included are reported.

Figure S1: Histograms of household vector counts by species for (A) *An. funestus*, (B) *An. gambiae* with N=1,724 total households, reprinted with permission from Hast, et al, 2019.[1]

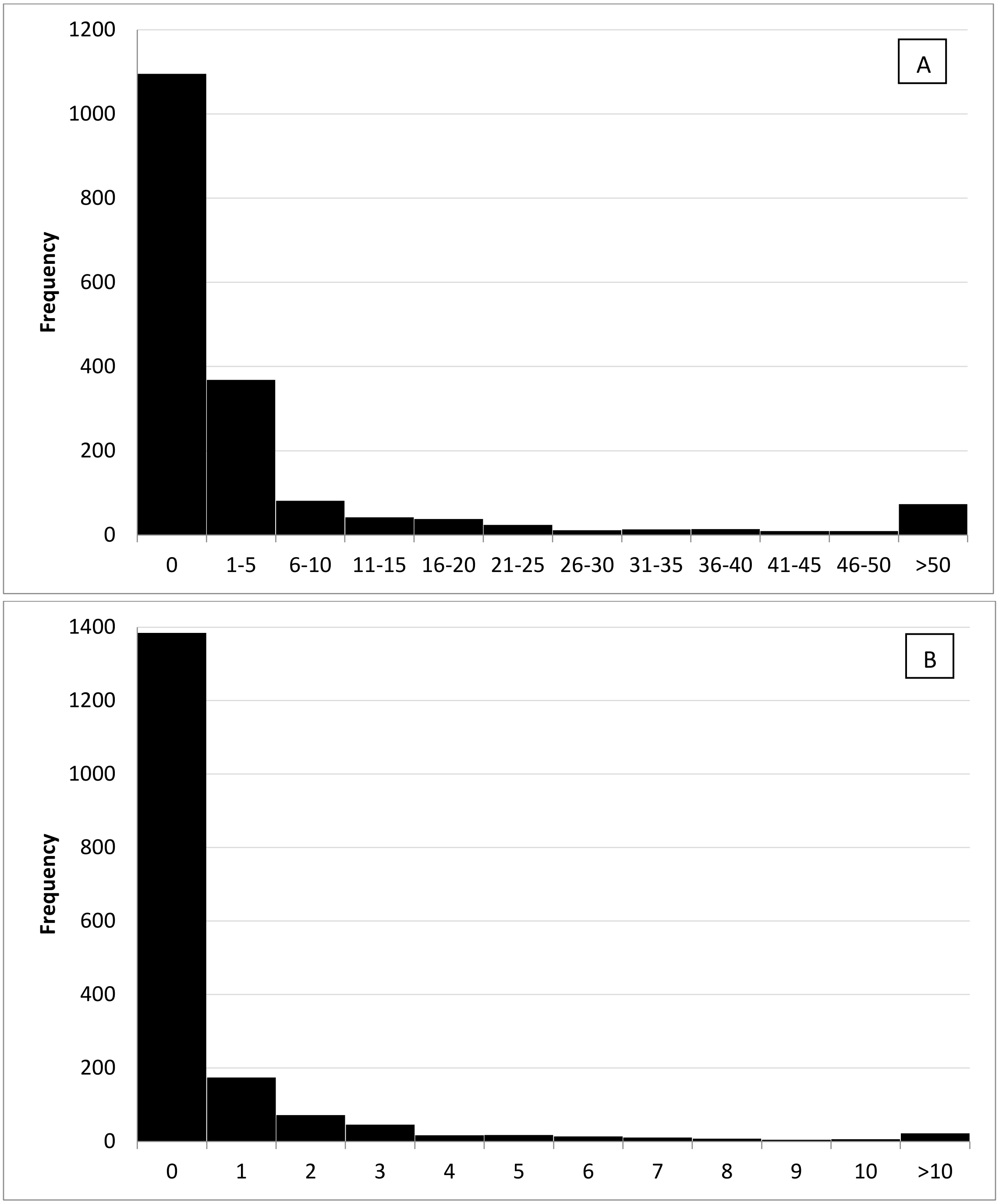

1. Hast, M.A., et al., *Risk Factors for Household Vector Abundance Using Indoor CDC Light Traps in a High Malaria Transmission Area of Northern Zambia.* Am J Trop Med Hyg, 2019. **101**(1): p. 126-136.

Figure S2: Adjusted and unadjusted percent reduction in vector densities by year compared to pre-IRS time period in A) *An. funestus* and B) *An. gambiae*

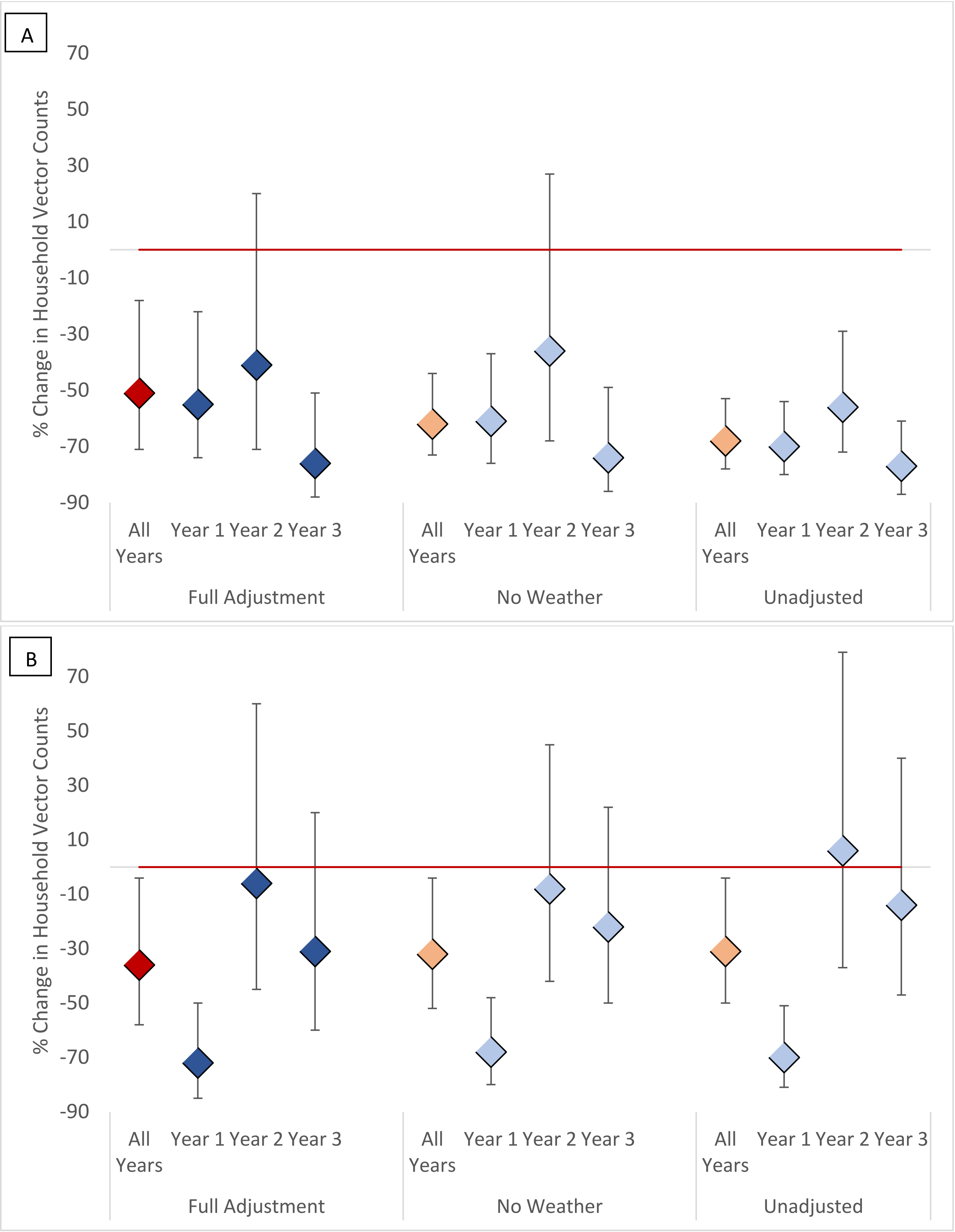

Table S1: Sensitivity analysis of impact of targeted IRS with pirimiphos-methyl on *An. funestus* and *An. gambiae* counts per household within the areas targeted for spraying, excluding counts ≥3 standard deviations above the mean, using negative binomial multivariate models, robust standard errors, and GEE clustered by household. *An. funestus* N=1246; *An. gambiae* N=1,249

	An. funestus			*An. gambiae*		
	IRR	95% CI	P value	IRR	95% CI	P value
Post-IRS	0.52	(0.34, 0.78)	*0.002*	0.72	(0.52, 1.00)	*0.05*
Open water source	-			-		
HH within 500 m (by 100 HH)	0.62	(0.56, 0.70)	*<0.001*	0.82	(0.76, 0.89)	*<0.001*
Elevation (by 10 m)	0.47	(0.37, 0.59)	*<0.001*	0.92	(0.84, 1.00)	*0.05*
Slope	0.82	(0.73, 0.91)	*0.001*	-		
NDVI (by 10%)	1.30	(1.00, 1.68)	*0.05*	-		
Distance from Lake Mweru (1000 in km)	1.16	(1.09, 1.24)	*<0.001*	-		
Distance from cat. 1 streams (in km)	0.58	(0.36, 0.94)	*0.03*	0.57	(0.39, 0.83)	*0.003*
Distance from cat. 4 streams (in km)	1.21	(1.03, 1.42)	*0.02*	-		
Lagged rainfall (by 10 mm) [1]	0.34	(0.21, 0.55)	*<0.001*	-		
Lagged rainfall (by 10 mm) [2]	-			3.71	(2.05, 6.70)	*<0.001*
Lagged maximum temperature (in C°) [3]	0.92	(0.83, 1.02)	0.1	-		
Lagged minimum temperature (in C°) [4]	-			1.09	(1.01, 1.18)	*<0.03*

PRR = prevalence rate ratio, CI = confidence interval, HOH = head of household, HH = household

[1] Interval=2 weeks, lag=2 weeks; [2] Interval=7 weeks, lag=5 weeks; [3] interval=8 weeks, lag=7 weeks; [4] interval=7 weeks, lag=2 weeks

Table S2: Sensitivity analysis of impact of targeted IRS with pirimiphos-methyl on *An. funestus* and *An. gambiae* counts per household within the areas targeted for spraying, excluding inland sprayed areas, using negative binomial multivariate models, robust standard errors, and GEE clustered by household, N=1,073

	An. funestus			*An. gambiae*		
	IRR	95% CI	P value	IRR	95% CI	P value
Post-IRS	0.49	(0.25, 0.94)	*0.03*	0.57	(0.37, 0.97)	*0.04*
Open water source	-			1.50	(1.03, 2.20)	*0.04*
HH within 500 m (by 100 HH)	0.59	(0.51, 0.68)	*<0.001*	0.79	(0.72, 0.87)	*<0.001*
Elevation (by 10 m)	0.43	(0.30, 0.62)	*<0.001*	-		
Slope	0.80	(0.68, 0.93)	*0.004*	0.90	(0.78, 1.03)	0.1
Distance from cat. 1 streams (in km)	0.39	(0.21, 0.73)	*0.003*	0.60	(0.38, 0.93)	*0.02*
Distance from cat. 4 streams (in km)	1.50	(1.14, 1.98)	*0.004*	-		
Lagged rainfall (by 10 mm) [1]	0.30	(0.13, 0.68)	*0.004*	-		
Lagged rainfall (by 10 mm) [2]	-			3.69	(1.54, 8.84)	*0.003*
Lagged maximum temperature (in C°) [3]	1.19	(1.05, 1.35)	*0.008*	-		
Lagged maximum temperature (in C°) [4]	0.75	(0.61, 0.92)	*0.007*	-		
Lagged maximum temperature (in C°) [5]	-			0.72	(0.59, 0.87)	*0.001*
Lagged minimum temperature (in C°) [6]	-			1.36	(1.19, 1.55)	*<0.001*

PRR = prevalence rate ratio, CI = confidence interval, HOH = head of household, HH = household

[1] Interval=2 weeks, lag=2 weeks; [2] Interval=9 weeks, lag=5 weeks; [3] interval=1 weeks, lag=2 weeks; [4] interval=8 weeks, lag=3 weeks; [5] interval=7 weeks, lag=4 weeks; [6] interval=9 weeks, lag=7 weeks
